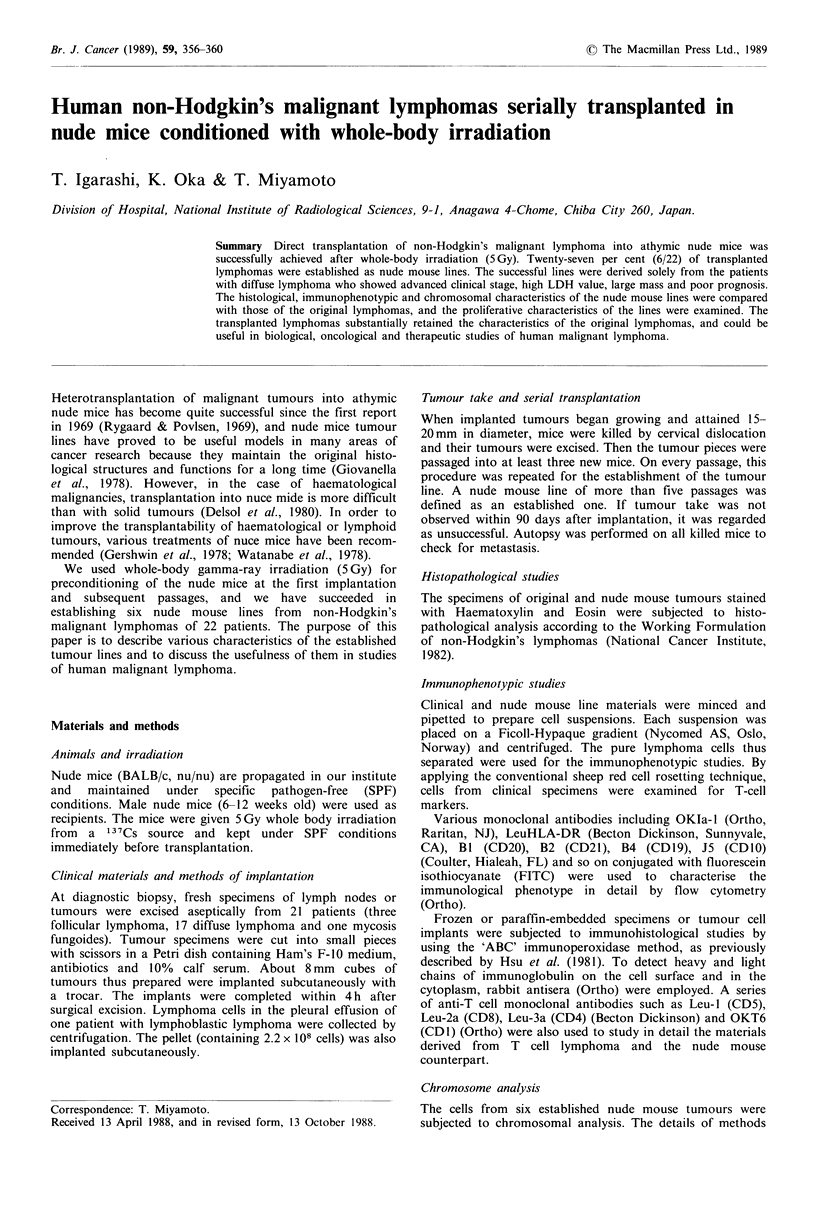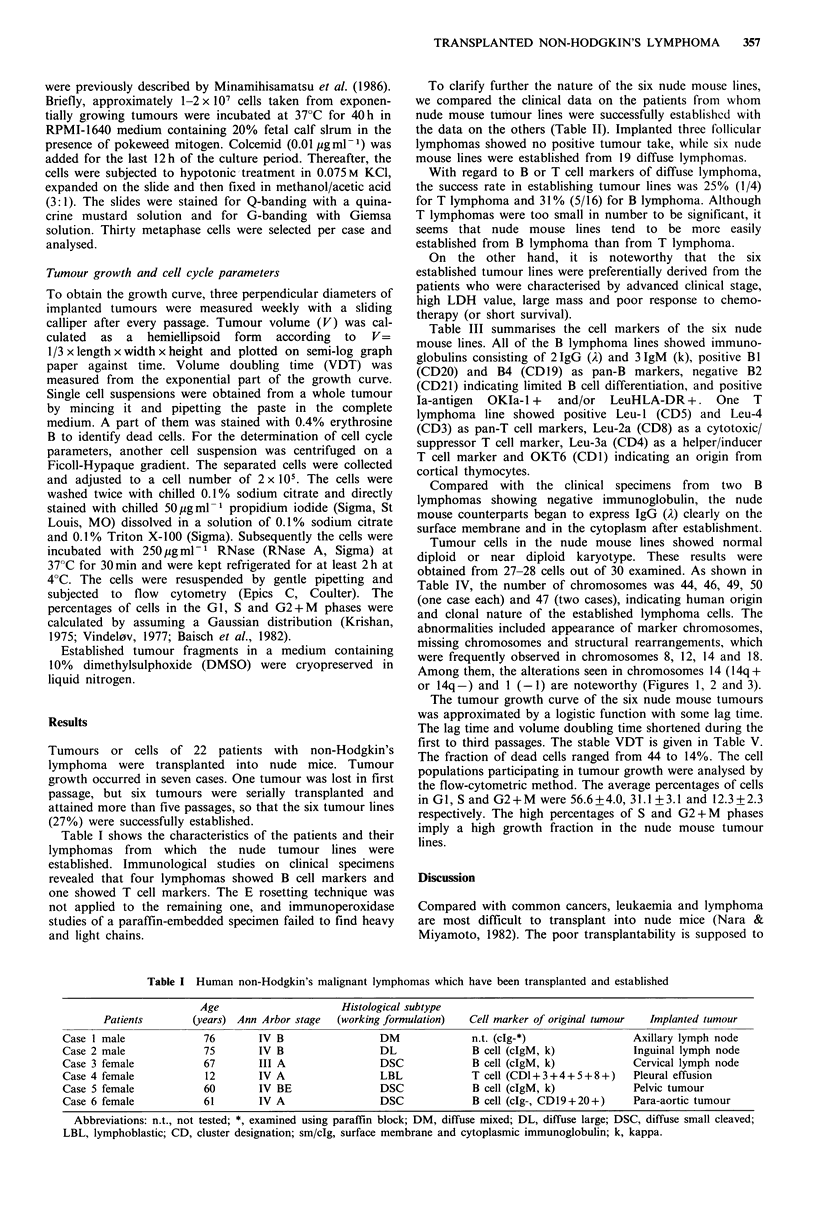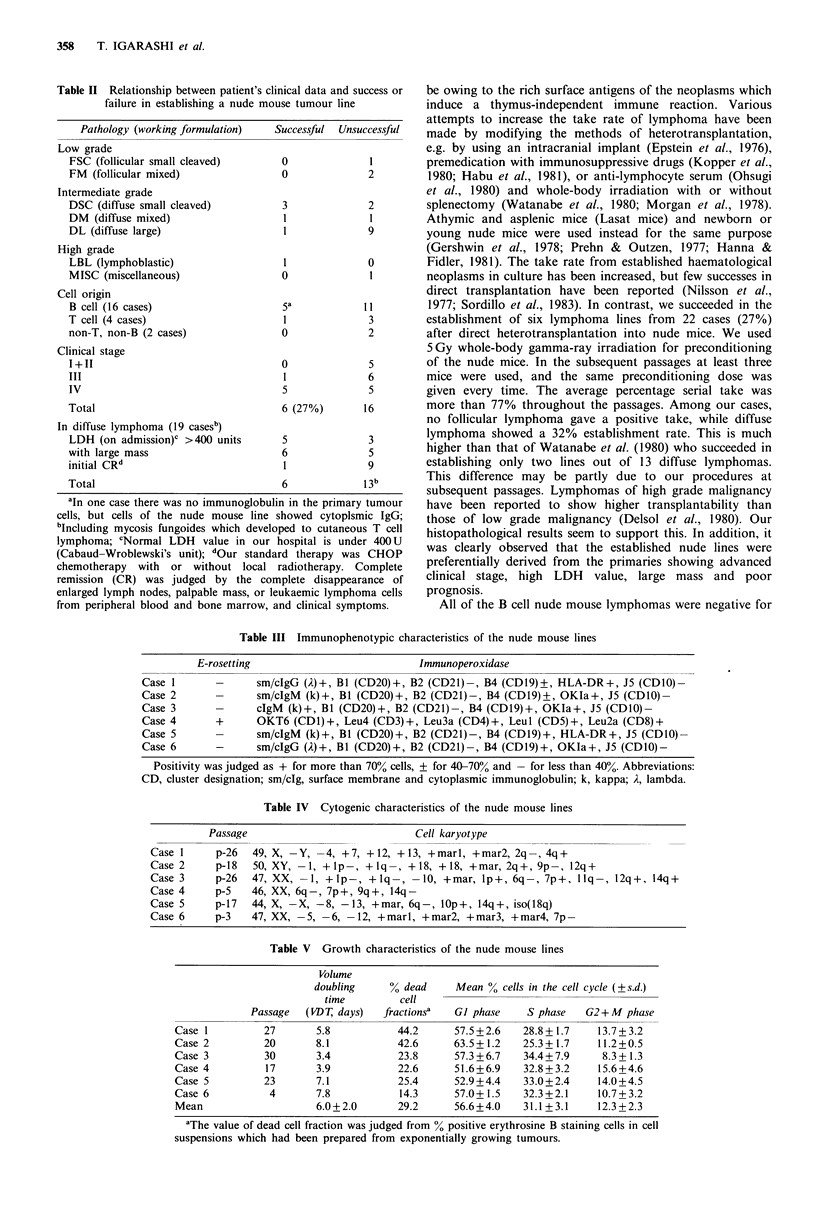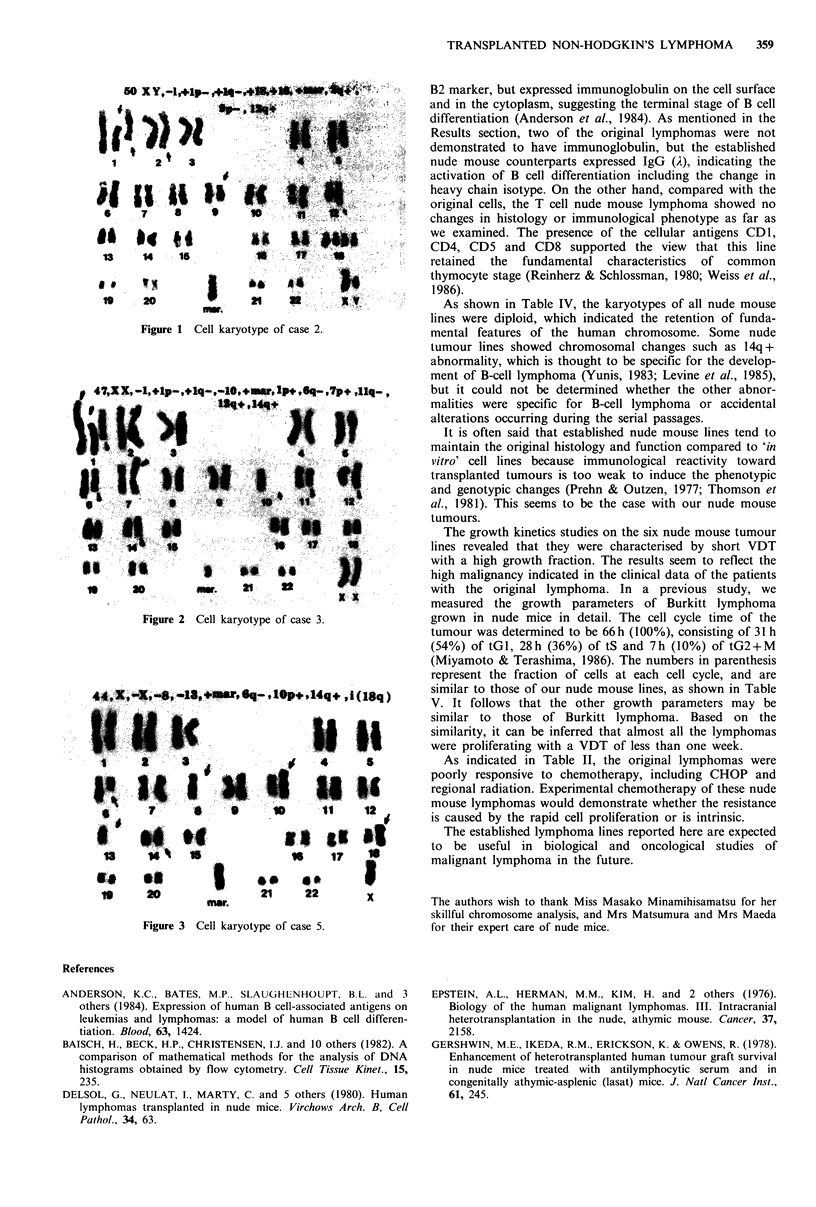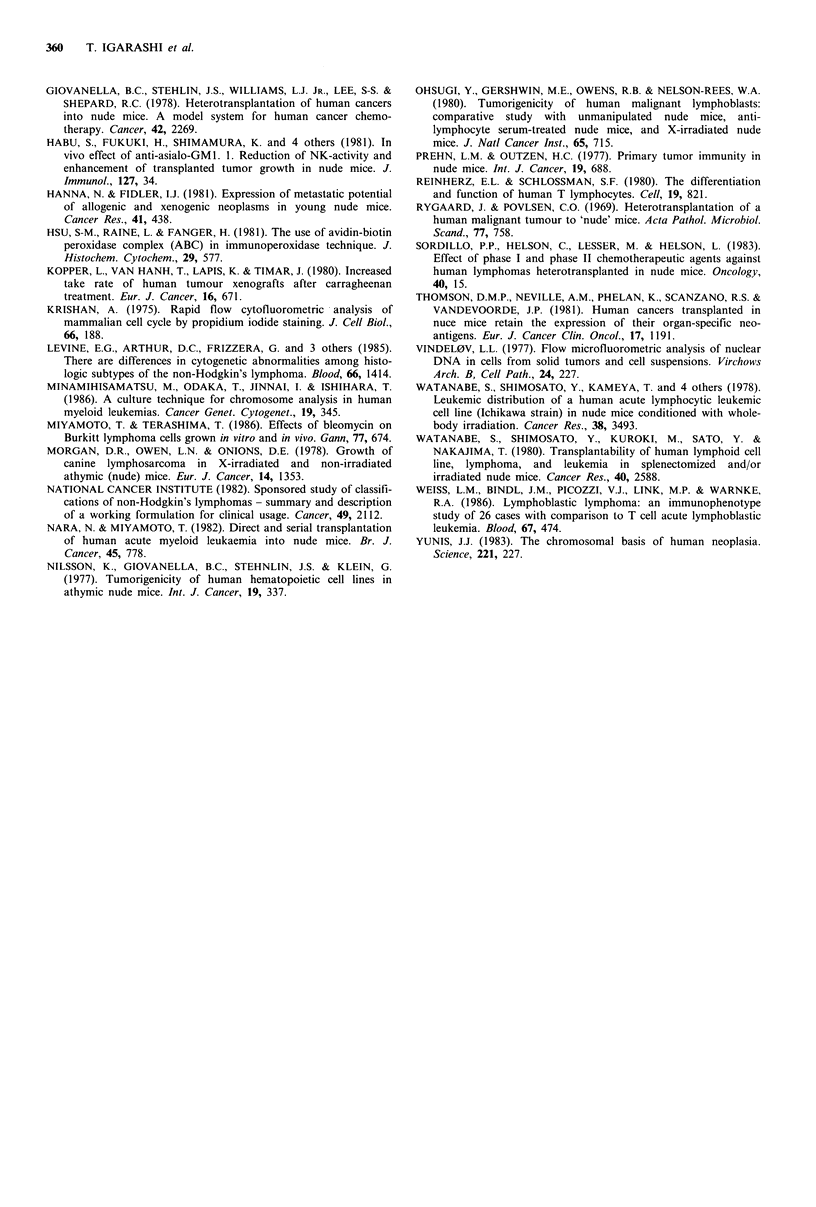# Human non-Hodgkin's malignant lymphomas serially transplanted in nude mice conditioned with whole-body irradiation.

**DOI:** 10.1038/bjc.1989.71

**Published:** 1989-03

**Authors:** T. Igarashi, K. Oka, T. Miyamoto

**Affiliations:** Division of Hospital, National Institute of Radiological Sciences, Chiba City, Japan.

## Abstract

**Images:**


Br. J. Cancer (1989), 59, 356–360 © The Macmillan Press Ltd., 1989

### Human non-Hodgkin's malignant lymphomas serially transplanted in nude mice conditioned with whole-body irradiation

T. Igarashi, K. Oka & T. Miyamoto

*Division of Hospital, National Institute of Radiological Sciences, 9-1, Anagawa 4-Chome, Chiba City 260, Japan.*

**Summary** Direct transplantation of non-Hodgkin's malignant lymphoma into athymic nude mice was successfully achieved after whole-body irradiation (5 Gy). Twenty-seven per cent (6/22) of transplanted lymphomas were established as nude mouse lines. The successful lines were derived solely from the patients with diffuse lymphoma who showed advanced clinical stage, high LDH value, large mass and poor prognosis. The histological, immunophenotypic and chromosomal characteristics of the nude mouse lines were compared with those of the original lymphomas, and the proliferative characteristics of the lines were examined. The transplanted lymphomas substantially retained the characteristics of the original lymphomas, and could be useful in biological, oncological and therapeutic studies of human malignant lymphoma.

Heterotransplantation of malignant tumours into athymic nude mice has become quite successful since the first report in 1969 (Rygaard & Povlsen, 1969), and nude mice tumour lines have proved to be useful models in many areas of cancer research because they maintain the original histological structures and functions for a long time (Giovanella *et al.*, 1978). However, in the case of haematological malignancies, transplantation into nuce mide is more difficult than with solid tumours (Delsol *et al.*, 1980). In order to improve the transplantability of haematological or lymphoid tumours, various treatments of nuce mice have been recommended (Gershwin *et al.*, 1978; Watanabe *et al.*, 1978).

We used whole-body gamma-ray irradiation (5 Gy) for preconditioning of the nude mice at the first implantation and subsequent passages, and we have succeeded in establishing six nude mouse lines from non-Hodgkin's malignant lymphomas of 22 patients. The purpose of this paper is to describe various characteristics of the established tumour lines and to discuss the usefulness of them in studies of human malignant lymphoma.

### Materials and methods

### *Animals and irradiation*

Nude mice (BALB/c, nu/nu) are propagated in our institute and maintained under specific pathogen-free (SPF) conditions. Male nude mice (6–12 weeks old) were used as recipients. The mice were given 5 Gy whole body irradiation from a $^{137}$Cs source and kept under SPF conditions immediately before transplantation.

### *Clinical materials and methods of implantation*

At diagnostic biopsy, fresh specimens of lymph nodes or tumours were excised aseptically from 21 patients (three follicular lymphoma, 17 diffuse lymphoma and one mycosis fungoides). Tumour specimens were cut into small pieces with scissors in a Petri dish containing Ham's F-10 medium, antibiotics and 10% calf serum. About 8 mm cubes of tumours thus prepared were implanted subcutaneously with a trocar. The implants were completed within 4 h after surgical excision. Lymphoma cells in the pleural effusion of one patient with lymphoblastic lymphoma were collected by centrifugation. The pellet (containing $2.2 \times 10^8$ cells) was also implanted subcutaneously.

Correspondence: T. Miyamoto.
Received 13 April 1988, and in revised form, 13 October 1988.

### *Tumour take and serial transplantation*

When implanted tumours began growing and attained 15–20 mm in diameter, mice were killed by cervical dislocation and their tumours were excised. Then the tumour pieces were passaged into at least three new mice. On every passage, this procedure was repeated for the establishment of the tumour line. A nude mouse line of more than five passages was defined as an established one. If tumour take was not observed within 90 days after implantation, it was regarded as unsuccessful. Autopsy was performed on all killed mice to check for metastasis.

### *Histopathological studies*

The specimens of original and nude mouse tumours stained with Haematoxylin and Eosin were subjected to histopathological analysis according to the Working Formulation of non-Hodgkin's lymphomas (National Cancer Institute, 1982).

### *Immunophenotypic studies*

Clinical and nude mouse line materials were minced and pipetted to prepare cell suspensions. Each suspension was placed on a Ficoll-Hypaque gradient (Nycomed AS, Oslo, Norway) and centrifuged. The pure lymphoma cells thus separated were used for the immunophenotypic studies. By applying the conventional sheep red cell rosetting technique, cells from clinical specimens were examined for T-cell markers.

Various monoclonal antibodies including OKIa-1 (Ortho, Raritan, NJ), LeuHLA-DR (Becton Dickinson, Sunnyvale, CA), B1 (CD20), B2 (CD21), B4 (CD19), J5 (CD10) (Coulter, Hialeah, FL) and so on conjugated with fluorescein isothiocyanate (FITC) were used to characterise the immunological phenotype in detail by flow cytometry (Ortho).

Frozen or paraffin-embedded specimens or tumour cell implants were subjected to immunohistological studies by using the 'ABC' immunoperoxidase method, as previously described by Hsu *et al.* (1981). To detect heavy and light chains of immunoglobulin on the cell surface and in the cytoplasm, rabbit antisera (Ortho) were employed. A series of anti-T cell monoclonal antibodies such as Leu-1 (CD5), Leu-2a (CD8), Leu-3a (CD4) (Becton Dickinson) and OKT6 (CD1) (Ortho) were also used to study in detail the materials derived from T cell lymphoma and the nude mouse counterpart.

### *Chromosome analysis*

The cells from six established nude mouse tumours were subjected to chromosomal analysis. The details of methods were previously described by Minamihisamatsu *et al.* (1986). Briefly, approximately 1–2 × $10^7$ cells taken from exponentially growing tumours were incubated at 37°C for 40 h in RPMI-1640 medium containing 20% fetal calf slrum in the presence of pokeweed mitogen. Colcemid (0.01 $\mu g\,ml^{-1}$) was added for the last 12 h of the culture period. Thereafter, the cells were subjected to hypotonic treatment in 0.075 M KCl, expanded on the slide and then fixed in methanol/acetic acid (3:1). The slides were stained for Q-banding with a quinacrine mustard solution and for G-banding with Giemsa solution. Thirty metaphase cells were selected per case and analysed.

*Tumour growth and cell cycle parameters*

To obtain the growth curve, three perpendicular diameters of implanted tumours were measured weekly with a sliding calliper after every passage. Tumour volume ($V$) was calculated as a hemiellipsoid form according to $V = 1/3 \times \text{length} \times \text{width} \times \text{height}$ and plotted on semi-log graph paper against time. Volume doubling time (VDT) was measured from the exponential part of the growth curve. Single cell suspensions were obtained from a whole tumour by mincing it and pipetting the paste in the complete medium. A part of them was stained with 0.4% erythrosine B to identify dead cells. For the determination of cell cycle parameters, another cell suspension was centrifuged on a Ficoll-Hypaque gradient. The separated cells were collected and adjusted to a cell number of $2 \times 10^5$. The cells were washed twice with chilled 0.1% sodium citrate and directly stained with chilled 50 $\mu g\,ml^{-1}$ propidium iodide (Sigma, St Louis, MO) dissolved in a solution of 0.1% sodium citrate and 0.1% Triton X-100 (Sigma). Subsequently the cells were incubated with 250 $\mu g\,ml^{-1}$ RNase (RNase A, Sigma) at 37°C for 30 min and were kept refrigerated for at least 2 h at 4°C. The cells were resuspended by gentle pipetting and subjected to flow cytometry (Epics C, Coulter). The percentages of cells in the G1, S and G2+M phases were calculated by assuming a Gaussian distribution (Krishan, 1975; Vindeløv, 1977; Baisch *et al.*, 1982).

Established tumour fragments in a medium containing 10% dimethylsulphoxide (DMSO) were cryopreserved in liquid nitrogen.

### Results

Tumours or cells of 22 patients with non-Hodgkin's lymphoma were transplanted into nude mice. Tumour growth occurred in seven cases. One tumour was lost in first passage, but six tumours were serially transplanted and attained more than five passages, so that the six tumour lines (27%) were successfully established.

Table I shows the characteristics of the patients and their lymphomas from which the nude tumour lines were established. Immunological studies on clinical specimens revealed that four lymphomas showed B cell markers and one showed T cell markers. The E rosetting technique was not applied to the remaining one, and immunoperoxidase studies of a paraffin-embedded specimen failed to find heavy and light chains.

To clarify further the nature of the six nude mouse lines, we compared the clinical data on the patients from whom nude mouse tumour lines were successfully established with the data on the others (Table II). Implanted three follicular lymphomas showed no positive tumour take, while six nude mouse lines were established from 19 diffuse lymphomas.

With regard to B or T cell markers of diffuse lymphoma, the success rate in establishing tumour lines was 25% (1/4) for T lymphoma and 31% (5/16) for B lymphoma. Although T lymphomas were too small in number to be significant, it seems that nude mouse lines tend to be more easily established from B lymphoma than from T lymphoma.

On the other hand, it is noteworthy that the six established tumour lines were preferentially derived from the patients who were characterised by advanced clinical stage, high LDH value, large mass and poor response to chemotherapy (or short survival).

Table III summarises the cell markers of the six nude mouse lines. All of the B lymphoma lines showed immunoglobulins consisting of 2 IgG (λ) and 3 IgM (k), positive B1 (CD20) and B4 (CD19) as pan-B markers, negative B2 (CD21) indicating limited B cell differentiation, and positive Ia-antigen OKIa-1+ and/or LeuHLA-DR+. One T lymphoma line showed positive Leu-1 (CD5) and Leu-4 (CD3) as pan-T cell markers, Leu-2a (CD8) as a cytotoxic/suppressor T cell marker, Leu-3a (CD4) as a helper/inducer T cell marker and OKT6 (CD1) indicating an origin from cortical thymocytes.

Compared with the clinical specimens from two B lymphomas showing negative immunoglobulin, the nude mouse counterparts began to express IgG (λ) clearly on the surface membrane and in the cytoplasm after establishment.

Tumour cells in the nude mouse lines showed normal diploid or near diploid karyotype. These results were obtained from 27–28 cells out of 30 examined. As shown in Table IV, the number of chromosomes was 44, 46, 49, 50 (one case each) and 47 (two cases), indicating human origin and clonal nature of the established lymphoma cells. The abnormalities included appearance of marker chromosomes, missing chromosomes and structural rearrangements, which were frequently observed in chromosomes 8, 12, 14 and 18. Among them, the alterations seen in chromosomes 14 (14q+ or 14q−) and 1 (−1) are noteworthy (Figures 1, 2 and 3).

The tumour growth curve of the six nude mouse tumours was approximated by a logistic function with some lag time. The lag time and volume doubling time shortened during the first to third passages. The stable VDT is given in Table V. The fraction of dead cells ranged from 44 to 14%. The cell populations participating in tumour growth were analysed by the flow-cytometric method. The average percentages of cells in G1, S and G2+M were 56.6±4.0, 31.1±3.1 and 12.3±2.3 respectively. The high percentages of S and G2+M phases imply a high growth fraction in the nude mouse tumour lines.

### Discussion

Compared with common cancers, leukaemia and lymphoma are most difficult to transplant into nude mice (Nara & Miyamoto, 1982). The poor transplantability is supposed to

**Table I** Human non-Hodgkin's malignant lymphomas which have been transplanted and established

| *Patients* | *Age (years)* | *Ann Arbor stage* | *Histological subtype (working formulation)* | *Cell marker of original tumour* | *Implanted tumour* |
|---|---|---|---|---|---|
| Case 1 male | 76 | IV B | DM | n.t. (cIg-*) | Axillary lymph node |
| Case 2 male | 75 | IV B | DL | B cell (cIgM, k) | Inguinal lymph node |
| Case 3 female | 67 | III A | DSC | B cell (cIgM, k) | Cervical lymph node |
| Case 4 female | 12 | IV A | LBL | T cell (CD1+3+4+5+8+) | Pleural effusion |
| Case 5 female | 60 | IV BE | DSC | B cell (cIgM, k) | Pelvic tumour |
| Case 6 female | 61 | IV A | DSC | B cell (cIg-, CD19+20+) | Para-aortic tumour |

Abbreviations: n.t., not tested; *, examined using paraffin block; DM, diffuse mixed; DL, diffuse large; DSC, diffuse small cleaved; LBL, lymphoblastic; CD, cluster designation; sm/cIg, surface membrane and cytoplasmic immunoglobulin; k, kappa.

**Table II** Relationship between patient's clinical data and success or failure in establishing a nude mouse tumour line

| *Pathology (working formulation)* | *Successful* | *Unsuccessful* |
|---|---|---|
| Low grade | | |
| FSC (follicular small cleaved) | 0 | 1 |
| FM (follicular mixed) | 0 | 2 |
| Intermediate grade | | |
| DSC (diffuse small cleaved) | 3 | 2 |
| DM (diffuse mixed) | 1 | 1 |
| DL (diffuse large) | 1 | 9 |
| High grade | | |
| LBL (lymphoblastic) | 1 | 0 |
| MISC (miscellaneous) | 0 | 1 |
| Cell origin | | |
| B cell (16 cases) | 5[a] | 11 |
| T cell (4 cases) | 1 | 3 |
| non-T, non-B (2 cases) | 0 | 2 |
| Clinical stage | | |
| I+II | 0 | 5 |
| III | 1 | 6 |
| IV | 5 | 5 |
| Total | 6 (27%) | 16 |
| In diffuse lymphoma (19 cases[b]) | | |
| LDH (on admission)[c] >400 units | 5 | 3 |
| with large mass | 6 | 5 |
| initial CR[d] | 1 | 9 |
| Total | 6 | 13[b] |

[a]In one case there was no immunoglobulin in the primary tumour cells, but cells of the nude mouse line showed cytoplsmic IgG; [b]Including mycosis fungoides which developed to cutaneous T cell lymphoma; [c]Normal LDH value in our hospital is under 400U (Cabaud–Wroblewski's unit); [d]Our standard therapy was CHOP chemotherapy with or without local radiotherapy. Complete remission (CR) was judged by the complete disappearance of enlarged lymph nodes, palpable mass, or leukaemic lymphoma cells from peripheral blood and bone marrow, and clinical symptoms.

be owing to the rich surface antigens of the neoplasms which induce a thymus-independent immune reaction. Various attempts to increase the take rate of lymphoma have been made by modifying the methods of heterotransplantation, e.g. by using an intracranial implant (Epstein *et al.*, 1976), premedication with immunosuppressive drugs (Kopper *et al.*, 1980; Habu *et al.*, 1981), or anti-lymphocyte serum (Ohsugi *et al.*, 1980) and whole-body irradiation with or without splenectomy (Watanabe *et al.*, 1980; Morgan *et al.*, 1978). Athymic and asplenic mice (Lasat mice) and newborn or young nude mice were used instead for the same purpose (Gershwin *et al.*, 1978; Prehn & Outzen, 1977; Hanna & Fidler, 1981). The take rate from established haematological neoplasms in culture has been increased, but few successes in direct transplantation have been reported (Nilsson *et al.*, 1977; Sordillo *et al.*, 1983). In contrast, we succeeded in the establishment of six lymphoma lines from 22 cases (27%) after direct heterotransplantation into nude mice. We used 5 Gy whole-body gamma-ray irradiation for preconditioning of the nude mice. In the subsequent passages at least three mice were used, and the same preconditioning dose was given every time. The average percentage serial take was more than 77% throughout the passages. Among our cases, no follicular lymphoma gave a positive take, while diffuse lymphoma showed a 32% establishment rate. This is much higher than that of Watanabe *et al.* (1980) who succeeded in establishing only two lines out of 13 diffuse lymphomas. This difference may be partly due to our procedures at subsequent passages. Lymphomas of high grade malignancy have been reported to show higher transplantability than those of low grade malignancy (Delsol *et al.*, 1980). Our histopathological results seem to support this. In addition, it was clearly observed that the established nude lines were preferentially derived from the primaries showing advanced clinical stage, high LDH value, large mass and poor prognosis.

All of the B cell nude mouse lymphomas were negative for

**Table III** Immunophenotypic characteristics of the nude mouse lines

| | *E-rosetting* | *Immunoperoxidase* |
|---|---|---|
| Case 1 | – | sm/cIgG (λ)+, B1 (CD20)+, B2 (CD21)–, B4 (CD19)±, HLA-DR+, J5 (CD10)– |
| Case 2 | – | sm/cIgM (k)+, B1 (CD20)+, B2 (CD21)–, B4 (CD19)±, OKIa+, J5 (CD10)– |
| Case 3 | – | cIgM (k)+, B1 (CD20)+, B2 (CD21)–, B4 (CD19)+, OKIa+, J5 (CD10)– |
| Case 4 | + | OKT6 (CD1)+, Leu4 (CD3)+, Leu3a (CD4)+, Leu1 (CD5)+, Leu2a (CD8)+ |
| Case 5 | – | sm/cIgM (k)+, B1 (CD20)+, B2 (CD21)–, B4 (CD19)+, HLA-DR+, J5 (CD10)– |
| Case 6 | – | sm/cIgG (λ)+, B1 (CD20)+, B2 (CD21)–, B4 (CD19)+, OKIa+, J5 (CD10)– |

Positivity was judged as + for more than 70% cells, ± for 40–70% and – for less than 40%. Abbreviations: CD, cluster designation; sm/cIg, surface membrane and cytoplasmic immunoglobulin; k, kappa; λ, lambda.

**Table IV** Cytogenic characteristics of the nude mouse lines

| | *Passage* | *Cell karyotype* |
|---|---|---|
| Case 1 | p-26 | 49, X, –Y, –4, +7, +12, +13, +mar1, +mar2, 2q–, 4q+ |
| Case 2 | p-18 | 50, XY, –1, +1p–, +1q–, +18, +18, +mar, 2q+, 9p–, 12q+ |
| Case 3 | p-26 | 47, XX, –1, +1p–, +1q–, –10, +mar, 1p+, 6q–, 7p+, 11q–, 12q+, 14q+ |
| Case 4 | p-5 | 46, XX, 6q–, 7p+, 9q+, 14q– |
| Case 5 | p-17 | 44, X, –X, –8, –13, +mar, 6q–, 10p+, 14q+, iso(18q) |
| Case 6 | p-3 | 47, XX, –5, –6, –12, +mar1, +mar2, +mar3, +mar4, 7p– |

**Table V** Growth characteristics of the nude mouse lines

| | *Passage* | *Volume doubling time (VDT, days)* | *% dead cell fractions*[a] | *Mean % cells in the cell cycle (±s.d.)* | | |
|---|---|---|---|---|---|---|
| | | | | *G1 phase* | *S phase* | *G2+M phase* |
| Case 1 | 27 | 5.8 | 44.2 | 57.5±2.6 | 28.8±1.7 | 13.7±3.2 |
| Case 2 | 20 | 8.1 | 42.6 | 63.5±1.2 | 25.3±1.7 | 11.2±0.5 |
| Case 3 | 30 | 3.4 | 23.8 | 57.3±6.7 | 34.4±7.9 | 8.3±1.3 |
| Case 4 | 17 | 3.9 | 22.6 | 51.6±6.9 | 32.8±3.2 | 15.6±4.6 |
| Case 5 | 23 | 7.1 | 25.4 | 52.9±4.4 | 33.0±2.4 | 14.0±4.5 |
| Case 6 | 4 | 7.8 | 14.3 | 57.0±1.5 | 32.3±2.1 | 10.7±3.2 |
| Mean | | 6.0±2.0 | 29.2 | 56.6±4.0 | 31.1±3.1 | 12.3±2.3 |

[a]The value of dead cell fraction was judged from % positive erythrosine B staining cells in cell suspensions which had been prepared from exponentially growing tumours.

**Figure 1** Cell karyotype of case 2.

**Figure 2** Cell karyotype of case 3.

**Figure 3** Cell karyotype of case 5.

B2 marker, but expressed immunoglobulin on the cell surface and in the cytoplasm, suggesting the terminal stage of B cell differentiation (Anderson *et al.*, 1984). As mentioned in the Results section, two of the original lymphomas were not demonstrated to have immunoglobulin, but the established nude mouse counterparts expressed IgG ($\lambda$), indicating the activation of B cell differentiation including the change in heavy chain isotype. On the other hand, compared with the original cells, the T cell nude mouse lymphoma showed no changes in histology or immunological phenotype as far as we examined. The presence of the cellular antigens CD1, CD4, CD5 and CD8 supported the view that this line retained the fundamental characteristics of common thymocyte stage (Reinherz & Schlossman, 1980; Weiss *et al.*, 1986).

As shown in Table IV, the karyotypes of all nude mouse lines were diploid, which indicated the retention of fundamental features of the human chromosome. Some nude tumour lines showed chromosomal changes such as 14q+ abnormality, which is thought to be specific for the development of B-cell lymphoma (Yunis, 1983; Levine *et al.*, 1985), but it could not be determined whether the other abnormalities were specific for B-cell lymphoma or accidental alterations occurring during the serial passages.

It is often said that established nude mouse lines tend to maintain the original histology and function compared to '*in vitro*' cell lines because immunological reactivity toward transplanted tumours is too weak to induce the phenotypic and genotypic changes (Prehn & Outzen, 1977; Thomson *et al.*, 1981). This seems to be the case with our nude mouse tumours.

The growth kinetics studies on the six nude mouse tumour lines revealed that they were characterised by short VDT with a high growth fraction. The results seem to reflect the high malignancy indicated in the clinical data of the patients with the original lymphoma. In a previous study, we measured the growth parameters of Burkitt lymphoma grown in nude mice in detail. The cell cycle time of the tumour was determined to be 66 h (100%), consisting of 31 h (54%) of tG1, 28 h (36%) of tS and 7 h (10%) of tG2+M (Miyamoto & Terashima, 1986). The numbers in parenthesis represent the fraction of cells at each cell cycle, and are similar to those of our nude mouse lines, as shown in Table V. It follows that the other growth parameters may be similar to those of Burkitt lymphoma. Based on the similarity, it can be inferred that almost all the lymphomas were proliferating with a VDT of less than one week.

As indicated in Table II, the original lymphomas were poorly responsive to chemotherapy, including CHOP and regional radiation. Experimental chemotherapy of these nude mouse lymphomas would demonstrate whether the resistance is caused by the rapid cell proliferation or is intrinsic.

The established lymphoma lines reported here are expected to be useful in biological and oncological studies of malignant lymphoma in the future.

The authors wish to thank Miss Masako Minamihisamatsu for her skillful chromosome analysis, and Mrs Matsumura and Mrs Maeda for their expert care of nude mice.

### References

ANDERSON, K.C., BATES, M.P., SLAUGHENHOUPT, B.L. and 3 others (1984). Expression of human B cell-associated antigens on leukemias and lymphomas: a model of human B cell differentiation. *Blood*, **63**, 1424.

BAISCH, H., BECK, H.P., CHRISTENSEN, I.J. and 10 others (1982). A comparison of mathematical methods for the analysis of DNA histograms obtained by flow cytometry. *Cell Tissue Kinet.*, **15**, 235.

DELSOL, G., NEULAT, I., MARTY, C. and 5 others (1980). Human lymphomas transplanted in nude mice. *Virchows Arch. B, Cell Pathol.*, **34**, 63.

EPSTEIN, A.L., HERMAN, M.M., KIM, H. and 2 others (1976). Biology of the human malignant lymphomas. III. Intracranial heterotransplantation in the nude, athymic mouse. *Cancer*, **37**, 2158.

GERSHWIN, M.E., IKEDA, R.M., ERICKSON, K. & OWENS, R. (1978). Enhancement of heterotransplanted human tumour graft survival in nude mice treated with antilymphocytic serum and in congenitally athymic-asplenic (lasat) mice. *J. Natl Cancer Inst.*, **61**, 245.

GIOVANELLA, B.C., STEHLIN, J.S., WILLIAMS, L.J. JR., LEE, S-S. & SHEPARD, R.C. (1978). Heterotransplantation of human cancers into nude mice. A model system for human cancer chemotherapy. *Cancer*, **42**, 2269.

HABU, S., FUKUKI, H., SHIMAMURA, K. and 4 others (1981). In vivo effect of anti-asialo-GM1. 1. Reduction of NK-activity and enhancement of transplanted tumor growth in nude mice. *J. Immunol.*, **127**, 34.

HANNA, N. & FIDLER, I.J. (1981). Expression of metastatic potential of allogenic and xenogenic neoplasms in young nude mice. *Cancer Res.*, **41**, 438.

HSU, S-M., RAINE, L. & FANGER, H. (1981). The use of avidin-biotin peroxidase complex (ABC) in immunoperoxidase technique. *J. Histochem. Cytochem.*, **29**, 577.

KOPPER, L., VAN HANH, T., LAPIS, K. & TIMAR, J. (1980). Increased take rate of human tumour xenografts after carragheenan treatment. *Eur. J. Cancer*, **16**, 671.

KRISHAN, A. (1975). Rapid flow cytofluorometric analysis of mammalian cell cycle by propidium iodide staining. *J. Cell Biol.*, **66**, 188.

LEVINE, E.G., ARTHUR, D.C., FRIZZERA, G. and 3 others (1985). There are differences in cytogenetic abnormalities among histologic subtypes of the non-Hodgkin's lymphoma. *Blood*, **66**, 1414.

MINAMIHISAMATSU, M., ODAKA, T., JINNAI, I. & ISHIHARA, T. (1986). A culture technique for chromosome analysis in human myeloid leukemias. *Cancer Genet. Cytogenet.*, **19**, 345.

MIYAMOTO, T. & TERASHIMA, T. (1986). Effects of bleomycin on Burkitt lymphoma cells grown *in vitro* and *in vivo*. *Gann*, **77**, 674.

MORGAN, D.R., OWEN, L.N. & ONIONS, D.E. (1978). Growth of canine lymphosarcoma in X-irradiated and non-irradiated athymic (nude) mice. *Eur. J. Cancer*, **14**, 1353.

NATIONAL CANCER INSTITUTE (1982). Sponsored study of classifications of non-Hodgkin's lymphomas – summary and description of a working formulation for clinical usage. *Cancer*, **49**, 2112.

NARA, N. & MIYAMOTO, T. (1982). Direct and serial transplantation of human acute myeloid leukaemia into nude mice. *Br. J. Cancer*, **45**, 778.

NILSSON, K., GIOVANELLA, B.C., STEHNLIN, J.S. & KLEIN, G. (1977). Tumorigenicity of human hematopoietic cell lines in athymic nude mice. *Int. J. Cancer*, **19**, 337.

OHSUGI, Y., GERSHWIN, M.E., OWENS, R.B. & NELSON-REES, W.A. (1980). Tumorigenicity of human malignant lymphoblasts: comparative study with unmanipulated nude mice, antilymphocyte serum-treated nude mice, and X-irradiated nude mice. *J. Natl Cancer Inst.*, **65**, 715.

PREHN, L.M. & OUTZEN, H.C. (1977). Primary tumor immunity in nude mice. *Int. J. Cancer*, **19**, 688.

REINHERZ, E.L. & SCHLOSSMAN, S.F. (1980). The differentiation and function of human T lymphocytes. *Cell*, **19**, 821.

RYGAARD, J. & POVLSEN, C.O. (1969). Heterotransplantation of a human malignant tumour to 'nude' mice. *Acta Pathol. Microbiol. Scand.*, **77**, 758.

SORDILLO, P.P., HELSON, C., LESSER, M. & HELSON, L. (1983). Effect of phase I and phase II chemotherapeutic agents against human lymphomas heterotransplanted in nude mice. *Oncology*, **40**, 15.

THOMSON, D.M.P., NEVILLE, A.M., PHELAN, K., SCANZANO, R.S. & VANDEVOORDE, J.P. (1981). Human cancers transplanted in nuce mice retain the expression of their organ-specific neoantigens. *Eur. J. Cancer Clin. Oncol.*, **17**, 1191.

VINDELØV, L.L. (1977). Flow microfluorometric analysis of nuclear DNA in cells from solid tumors and cell suspensions. *Virchows Arch. B, Cell Path.*, **24**, 227.

WATANABE, S., SHIMOSATO, Y., KAMEYA, T. and 4 others (1978). Leukemic distribution of a human acute lymphocytic leukemic cell line (Ichikawa strain) in nude mice conditioned with wholebody irradiation. *Cancer Res.*, **38**, 3493.

WATANABE, S., SHIMOSATO, Y., KUROKI, M., SATO, Y. & NAKAJIMA, T. (1980). Transplantability of human lymphoid cell line, lymphoma, and leukemia in splenectomized and/or irradiated nude mice. *Cancer Res.*, **40**, 2588.

WEISS, L.M., BINDL, J.M., PICOZZI, V.J., LINK, M.P. & WARNKE, R.A. (1986). Lymphoblastic lymphoma: an immunophenotype study of 26 cases with comparison to T cell acute lymphoblastic leukemia. *Blood*, **67**, 474.

YUNIS, J.J. (1983). The chromosomal basis of human neoplasia. *Science*, **221**, 227.